# Transcriptomic biomarkers of the response of hospitalized geriatric patients with infectious diseases

**DOI:** 10.1186/1742-4933-7-9

**Published:** 2010-08-17

**Authors:** Thi Kim Duy Vo, Patrice Godard, Marie de Saint-Hubert, Gabriel Morrhaye, Christian Swine, Vincent Geenen, Henri J Martens, Florence Debacq-Chainiaux, Olivier Toussaint

**Affiliations:** 1 Unit of Research on Cellular Biology, NARILIS-Namur Research Institute for Life Sciences, University of Namur (FUNDP), Rue de Bruxelles 61, B-5000 Namur, Belgium; 2 Department of Geriatrics University Hospital of Mont-Godinne, NARILIS-Namur Research Institute for Life Sciences, Université Catholique de Louvain (UCL), Av Dr G.Therasse, 1 B-5530, Yvoir, Belgium; 3 University of Liege, Center of Immunology, Laboratory of Immunoendocrinology, Institute of Pathology CHU-B23, B-4000 Liege-Sart Tilman, Belgium

## Abstract

**Background:**

Infectious diseases are significant causes of morbidity and mortality among elderly populations. However, the relationship between oxidative stress, immune function and inflammatory response in acute phase of the infectious disease is poorly understood.

**Results:**

Herein the abundance of a selection of 148 transcripts involved in immunosenescence and stress response was compared in total RNA of PBMC of 28 healthy aged probands and 39 aged patients in acute phase of infectious disease (day 2-4 after hospitalization) or in convalescence phase (day 7-10). This study provides a list of 24 differentially abundant transcript species in the acute phase versus healthy aged. For instance, transcripts associated with inflammatory and anti-inflammatory reactions (TNFRSF1A, IL1R1, IL1R2, IL10RB) and with oxidative stress (HMOX1, GPX1, SOD2, PRDX6) were more abundant while those associated with T-cell functions (CD28, CD69, LCK) were less abundant in acute phase. The abundance of seven of these transcripts (CD28, CD69, LCK, CTSD, HMOX1, TNFRSF1A and PRDX6) was already known to be altered in healthy aged probands compared to healthy young ones and was further affected in aged patients in acute phase, compromising an efficient response.

**Conclusion:**

This work provides insights of the state of acute phase response to infections in elderly patients and could explain further the lack of appropriate response in the elderly compared to younger persons.

## Introduction

Ageing is defined as a progressive deterioration of biological functions and physiological capacity, which leads to the accumulation of disabilities and diseases [1] with reduced ability to cope with environmental stimuli, dysregulated inflammatory responses and changes of the immune response. Immunosenescence refers to the gradual age-related deterioration or modification of the immune system [2]. This results in greater susceptibility to the risk of infectious diseases, inflammatory disorders, cancer and autoimmune diseases [3,4]. Immunosenescence is involved in the poor response of elderly to vaccination against pathogens such as influenza or pneumonia [5-7]. Ageing is associated with increased levels of circulating inflammatory components and chronic pro-inflammatory state, so called inflammageing [8], due to continuous antigenic stress that impinges upon innate immunity, throughout life, and has potential implications for the onset of inflammatory diseases [9]. While inflammation is an adaptive response to acute illness or injury, resulting in clearance of pathogens, inflammageing can be detrimental to health and be predisposing factor for age-related diseases. A higher level of pro-inflammatory cytokines is associated with a number of age-related diseases and/or with the development of frailty [10,11].

Infectious diseases are significant causes of morbidity and mortality among elderly population. Older persons generally have greater susceptibility to infections than younger adults [12]. This is particularly true with intracellular micro-organisms whose immunity is mostly cell-mediated. It is well known that ageing is associated with immunosenescence. However, other factors probably contribute to the greater susceptibility of infection and its adverse impact on host response in the elderly persons. Here, we used a transcriptomic approach to identify "signature genes" of acute phase response to infections in the peripheral blood mononuclear cell (PBMC) of the elderly persons. Using a designed gene expression micro-array, we reported the data analysis of samples collected from healthy aged probands and aged infectious patients during their acute and convalescence phases. The purpose of this study was to investigate insights of oxidative stress, immune function and inflammatory responses in acute phase response to infections in elderly. Hence, we developed a low-density DNA array to study the relative abundance of transcripts species involved in immunosenescence, inflammation and stress response, mainly based on the literature and as described in [13]. Inter- and intra-platform reproducibility of gene expression measurements was demonstrated previously with this technology [14,15]. Numerous verifications with real time retrotranscription quantitative polymerase chain reaction (RT-qPCR) have been done in different studies on senescence [16]. Verifications of results obtained with these arrays in very similar conditions using samples of total RNA from PBMC of old and young probands were also done [13].

## Methods

The recruitment was conducted at the University Hospital of Mont-Godinne (UCL, Belgium). Two groups were considered: aged healthy volunteers considered as control group and aged patients with infectious diseases. Healthy aged participants were recruited on a voluntary basis from different senior associations. To be eligible, participants needed: 1) to be aged of 75 years and over; 2) not to be institutionalised; 3) to have no evidence in the previous month of an acute medical condition, nor deterioration of a chronic condition. The aged patients with infectious diseases were older than 75 years and were hospitalized through the emergency department, with documented infection (bacteriological and/or radiological proofs), including pneumonia, diverticulitis, septicaemia or cellulitis. Patients were excluded if they used steroidal or nonsteroidal anti-inflammatory drugs one week before the inclusion (low doses of aspirin for cardiovascular prevention were tolerated), had active cancer or a previous hospital stay within the 2 previous weeks, were admitted for intensive or palliative care or were completely dependent in activities of daily living (ADL). The same aged infectious patients were evaluated and blood was taken during acute phase (defined as day 2 to 4 after admission) and convalescence phase (day 7 to 10). Moreover, in the same study, we recruited 25 healthy young probands, as previously described [13]. Their mean age was 35.0 ± 6.5 year old. This study was approved by the Ethical Committee of the hospital. Informed consent was obtained from all volunteers.

### 2) Plasma, isolation of PBMC, RNA extraction and integrity

After blood samples (16 ml) were collected, blood parameters including cell counts and assays of acute phase proteins: C-reactive protein (CRP), albumin, prealbumin, fibrinogen, D-dimers were routinely determined. The levels of interleukin-6 (IL-6), interleukin-10 (IL-10), tumor necrosis factor-alpha (TNF-α) were essayed by ELISA (Biosource, Belgium). PBMC were isolated by centrifugation on Ficoll-Hypaque gradient centrifugation (Becton Dickinson Vacutainer CPT, USA). Plasma was collected and stored at -80°C until analysis.

Cells were washed twice in HBSS buffer. After adjustment at 10^8 ^cells/ml, part of the suspension was dispatched for hemogram determination (ADVIA 120, Baker, USA). The rest was used for extraction of total RNA (Total RNAgents^® ^Total RNA Isolation System, Promega, USA) according to the manufacturer's protocol and stored at -80°C. After extraction of total RNA, the integrity of RNA was assessed (Agilent 2100 Bioanalyzer Agilent Technologies, Germany). Reverse transcription, RNA amplification and labelling using the MessageAmp™ II-Biotin Enhanced kit (Ambion, USA) were performed from 250 ng of total RNA.

### 3) Low density DNA-array analysis

#### Design of the array

We purchased a low-density DNA array "on-demand" able to detect 148 different transcript species (Eppendorf, Germany) involved in immunosenescence, and more specifically focusing the T-cell compartment, inflammation and oxidative stress, as previously described [13].

The system has two assays per glass side with three identical sub-arrays per assay. The sequences of the DNA covalently linked to the glass slide were carefully chosen by sequence comparison. Checks were made to ensure that no cross-hybridization takes place. Several positive and negative hybridization controls plus detection controls were spotted on the array in order to control the reliability of the experimental data.

#### Hybridization and detection

Hybridization on the array was carried out as described by the manufacturer using 15 μg of complementary DNA. The hybridization reaction was performed overnight at 60°C in a Thermoblock for DC (DualChip^®^) Slides used with a Thermomixer comfort (Eppendorf, Germany).

Detection was performed using a Cy3-conjugated IgG anti-biotin (Jackson Immuno Research Laboratories, USA). Fluorescence of the hybridized arrays was scanned using the Packard ScanArray (PerkinElmer, USA) at a resolution of 10 μm. To maximize the dynamic range of detection, the same arrays were scanned at three photomultiplier (PMT) gains for quantifying high- and low-copy expressed genes. The scanned 16-bit images were imported into the ImaGene 4.1 software (BioDiscovery, USA). The fluorescence intensity of each DNA spot (median intensity of each pixel present within the spot) was calculated using local median background subtraction. Intensity values from high-PMT-gain pictures were used, except in the case of saturated spots. In the latter case, intensity values from intermediate PMT-gain pictures (or from low PMT-gain pictures in case of spots also saturated in the intermediate PMT-gain pictures) were used after scale correction. Only spots with median intensity after background subtraction at least 2 fold higher than their local background were taken into account. The median of the three intensity values of the triplicate DNA spots was used in further steps.

#### Data normalization and inter-batch correction

All micro-arrays results were normalized against a single reference applying a local weighted regression [17]. The micro-arrays were provided in four batches. A systematic intensity bias was detected between each batch and was corrected successfully. Four samples were hybridized four times onto arrays of the four different batches. For each spotted sequence, a correction factor between the first batch and each of the others was computed using these four samples. This spot and batch dependent correction factor was applied to normalize intensities of each array. These normalized and corrected intensities were used in further statistical analyses as in [13].

### 4) Statistical analysis

The Mann Whitney test was used to compare the difference between the healthy participant and the patients with infectious diseases groups. Comparisons between acute and convalescence phase were performed using Wilcoxon's test. Data are expressed as mean ± SD. Statistically differentially abundant transcripts were selected. We used p-values with the Benjamini correction (P-value × m/g, where m is the number of variables and g the rank of the variable according to p-value) and Benjamini < 0.05.

## Results

The study involved 28 healthy volunteers, representing the control group (mean age 82.3 ± 6.0, range 75 to 103). The 39 aged patients suffered from infectious diseases, as described in materials and methods (mean age 81.8 ± 5.4, range 75 to 97). The two groups were homogenous with regard to age and gender. Thirty-three of the patients in acute phase (day 2-4) were followed until convalescence phase (day 7-10) when samples of blood were taken again.

### 1) Indicators of the acute-phase response

Serum levels of albumin, pre-albumin and number of lymphocytes were significantly lower in blood of aged patients with infectious diseases in acute phase compared to the control healthy aged probands (Table 1). The levels of IL-6, IL-10, TNF-α, CRP, fibrinogen, D-dimer were significantly higher, compared with the control group. During convalescence phase, intermediate levels were observed between the control group and the aged patients with infectious diseases in acute phase (Table 1). Number of lymphocytes was higher in convalescence phase than in acute phase. Serum levels of CRP, D-dimer, fibrinogen and IL-6 were lower in convalescence phase than in acute phase. The total leukocyte population and neutrophil numbers in blood were higher in acute and convalescence phases compared to control healthy aged group, with the highest values at acute phase. The lymphocytes count was significantly decreased in acute phase. The changes of serum levels of CRP, D-dimer, fibrinogen, IL-6 and numbers of lymphocytes, leukocytes, neutrophils were significantly different between acute and convalescence phases. Thus, the main indicators of acute phase were observed in the aged patients with infectious diseases recruited at day 2-4 after hospitalization.

**Table 1:** Cell counts, plasma concentration of acute phase proteins and pro-inflammatory cytokines.

				Benjamini		
				
	HA (m±SD)	AIA (m±SD)	AIC (m±SD)	AIA vs AIC	AIA vs HA	AIC vs HA
Cell counts (nb/μL)						
Lymphocytes	1531.76 ± 464.12	1086.58 ± 417.70	1442.65 ± 459.67	p < 0.001	p < 0.001	p < 0.001
Leucocytes	5975.76 ± 998.45	10601.16 ± 4623.98	8083.78 ± 2379.13	p < 0.001	p < 0.001	p < 0.001
Neutrophils	3762.94 ± 891.50	8734.72 ± 4284.35	5702.68 ± 2040.11	p < 0.001	p < 0.001	p < 0.001
						
Acute phase proteins						
Albumin (mg/dL)	4270.67 ± 443.31	2982.42 ± 483.71	ND		p < 0.001	
Prealbumin (mg/dL)	26.00 ± 4.74	10.69 ± 4.61	ND		p < 0.001	
CRP (mg/dL)	0.26 ± 0.41	18.34 ± 9.24	2.50 ± 3.45	p < 0.001	p < 0.001	p < 0.001
D-dimer (ng/mL)	647.22 ± 430.54	2442.90 ± 1692.26	1838.14 ± 1125.11	p < 0.05	p < 0.001	p < 0.05
Fibrinogen (mg/dL)	392.81 ± 65.54	752.09 ± 242.97	586.22 ± 172.04	p < 0.01	p < 0.001	p < 0.005
						
Pro-inflammatory cytokines						
IL6 (pg/mL)	14.01 ± 26.95	121.84 ± 98.73	56.28 ± 71.23	p < 0.001	p < 0.005	p < 0.001
IL10 (pg/mL)	1.15 ± 3.67	17.95 ± 109.00	ND		p < 0.001	
TNF alpha (pg/mL)	22.80 ± 12.57	52.30 ± 45.88	ND		p < 0.005	

Values are expressed as mean ± SD. CRP: C-reactive protein; ND: Not Determined; Healthy Aged (HA); Aged patients with Infectious diseases in Acute phase (AIA); Aged patients with Infectious diseases in Convalescence phase (AIC).

### 2) Identification of differentially abundant transcripts in acute phase

The low-density DNA array used in this study was composed 148 represented genes involved in immunosenescence, inflammation, and stress response, mainly selected from literature (list of these transcripts in Additional file 1, Table S1).

From the 148 transcript species including those of 13 housekeeping genes, 82 transcript species were detected in the samples of PBMC of all the subjects, 15 transcript species were not detectable in any of the subjects and 51 were not quantifiable. The 82 detected transcript species were thus eligible for further analysis. There was no situation where a transcript was detected in a condition and not detected in the other. Validation of the results obtained with these arrays by RT-qPCR was described previously in the same conditions of blood sampling, RNA collection and hybridization on these arrays [13].

To elucidate differences of the abundance of PBMC transcripts between aged patients at acute phase and healthy aged subjects, the results were analysed individually for the 82 eligible transcripts, using a Mann-Whitney test, with a Benjamini and Hochberg false-discovery rate, and a p-value < 0.05. This resulted in a list of 24 differentially abundant transcripts with 15 increases and 9 decreases. The list of these transcripts is shown in Table 2. From these transcripts, those for which abundance decreased drastically were EGR3, CD69, LCK, BCL2 and CD28, while IL1R2, SERPINB2, CLU and TIMP1 increased by more than 1.7 fold (Table 2). The abundance of these 24 transcripts was compared between acute and convalescence phases.

**Table 2:** Genes differentially expressed in PMBC of Aged patients with Infectious diseases in Acute phase (AIA) patients (n = 39) versus healthy aged (HA) probands (n = 28).

Gene Symbol	Gene Name	Accession Number	AIA/HA ratio	Benjamini
BCL2	B-cell CLL/lymphoma 2	NM_000633.1	0.58	p < 0.001
CASP8	Caspase 8, apoptosis-related cysteine peptidase	X98172.1	0.66	p < 0.001
CCL5	Chemokine (C-C motif) ligand 5	NM_002985.1	0.71	p < 0.001
CD28	CD28 antigen (Tp44)	NM_006139.1	0.61	p < 0.005
CD69	CD69 antigen (p60, early T-cell activation antigen)	NM_001781.1	0.44	p < 0.001
DDIT3	DNA-damage-inducible transcript 3	S40706.1	0.76	p < 0.01
DNAJB1	DnaJ (Hsp40) homolog, subfamily B, member 1	D49547.1	0.76	p < 0.001
EGR3	Early growth response 3	NM_004430.1	0.42	p < 0.05
LCK	Lymphocyte-specific protein tyrosine kinase	NM_005356.2	0.58	p < 0.001
				
CLU	Clusterin	J02908.1	1.79	p < 0.001
CTSD	Cathepsin D (lysosomal aspartyl peptidase)	M11233.1	1.50	p < 0.001
CTSZ	Cathepsin Z	AF136273.1	1.33	p < 0.05
GPX1	Glutathione peroxidase 1	M21304.1	1.21	p < 0.05
HMOX1	Heme oxygenase (decycling) 1	NM_002133.1	1.57	p < 0.001
IL10RB	Interleukin 10 receptor, beta	NM_000628.3	1.39	p < 0.001
IL1R1	Interleukin 1 receptor, type I	NM_000877.2	1.48	p < 0.05
IL1R2	Interleukin 1 receptor, type II	U74649.1	2.34	p < 0.001
PRDX6	Peroxiredoxin 6	NM_004905.1	1.23	p < 0.005
PSMD1	Proteasome (prosome, macropain) 26 S subunit, non-ATPase, 1	NM_002807.1	1.24	p < 0.001
SERPINB2	Serpin peptidase inhibitor, clade B (ovalbumin), member 2	J02685.1	2.17	p < 0.005
SOD2	Superoxide dismutase 2, mitochondrial	NM_000636.1	1.53	p < 0.001
TIMP1	TIMP metallopeptidase inhibitor 1	NM_003254.1	1.71	p < 0.001
TIMP2	TIMP metallopeptidase inhibitor 2	NM_003255.2	1.59	p < 0.001
TNFRSF1A	Tumor necrosis factor receptor superfamily, member 1A	NM_001065.2	1.45	p < 0.001

Statistical analysis was carried out with the Mann-Whitney test.

### 3) Comparison between acute and convalescence phase

**Figure 1:**
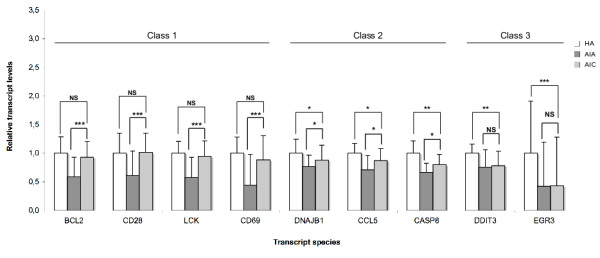
**Transcript species with decreased abundance in PMBC of Aged patients with Infectious diseases in Acute phase (AIA, n = 39), compared with healthy aged (HA, n = 28) probands and with Aged patients with Infectious diseases in Convalescence phase (AIC, n = 33)**. Transcripts are sorted according to 3 classes whether their abundance came back to the values of the control (healthy aged) at convalescence (class 1), significantly changed towards the values of the control at convalescence without full return to the values control (class 2) or not (class 3). Results are given as means ± standard deviation. Statistical analysis were carried out by the Mann-Whitney test for comparison between AIA and HA or AIC and HA, and by Wilcoxon's test for comparison between AIC and AIA. NS: Non significant (p ≥ 0.05); * p < 0.05; ** p < 0.01; *** p < 0.001.

**Figure 2:**
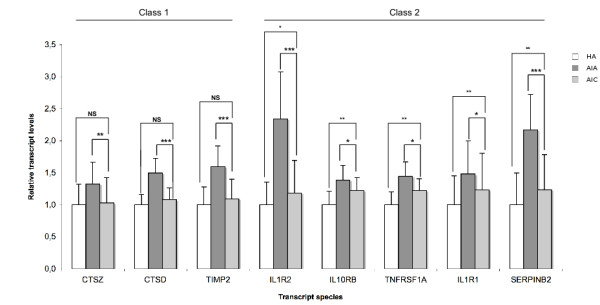
**Transcript species with increased abundance in PMBC of Aged patients with Infectious diseases in Acute phase (AIA, n = 39), compared with healthy aged (HA, n = 28) probands and with Aged patients with Infectious diseases in Convalescence phase (AIC, n = 33)**. Transcripts are sorted according to 3 classes whether their abundance came back to the values of the control (healthy aged) at convalescence (class 1, Figure 2), significantly changed towards the values of the control at convalescence without full return to the values control (class 2, Figure 2 right and 3 left) or not (class 3, Figure 3 right). Results are given as means ± standard deviation. Statistical analysis were carried out by the Mann-Whitney test for comparison between AIA and HA or AIC and HA, and by Wilcoxon's test for comparison between AIC and AIA. NS: Non significant (p ≥ 0.05); * p < 0.05; ** p < 0.01; *** p < 0.001.

**Figure 3:**
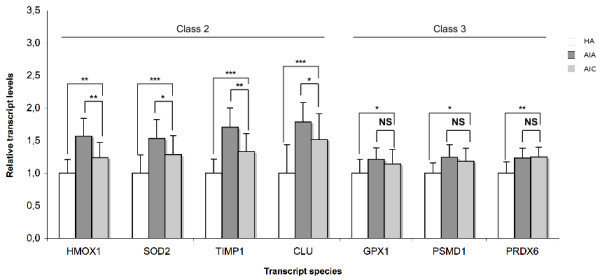
**Transcript species with increased abundance in PMBC of Aged patients with Infectious diseases in Acute phase (AIA, n = 39), compared with healthy aged (HA, n = 28) probands and with Aged patients with Infectious diseases in Convalescence phase (AIC, n = 33)**. Transcripts are sorted according to 3 classes whether their abundance came back to the values of the control (healthy aged) at convalescence (class 1, Figure 2), significantly changed towards the values of the control at convalescence without full return to the values control (class 2, Figure 2 right and 3 left) or not (class 3, Figure 3 right). Results are given as means ± standard deviation. Statistical analysis were carried out by the Mann-Whitney test for comparison between AIA and HA or AIC and HA, and by Wilcoxon's test for comparison between AIC and AIA. NS: Non significant (p ≥ 0.05); * p < 0.05; ** p < 0.01; *** p < 0.001.

At convalescence phase, one could sort out 3 classes of transcripts among the those for which abundance decreased in acute phase. Class 1 transcripts (BCL2, CD28, CD69 and LCK) came back to the control values of the healthy aged. In class 2 (CASP8, CCL5 and DNAJB1), there was a significant but incomplete return to the control value, which was not observed for class 3 (DDIT3 and EGR3, Fig 1). Following a similar rationale, the 15 transcripts whose abundance was increased in acute phase could be sorted in 3 classes at convalescence phase (Fig 2, 3). Class 1 transcripts returned to the control values of healthy aged (CTSD, CTSZ, TIMP2, Fig 2). In class 2 (CLU, HMOX1, IL10RB, IL1R1, IL1R2, SERPINB2, SOD2, TIMP1, TNFRSF1A), the abundance of the transcripts significantly decreased but did not return to control values (Fig 2, 3). In class 3 the abundance of the transcripts did not significantly decrease at convalescence (GPX1, PRDX6 and PSMD1, Fig 3).

## Discussion

The elderly population is particularly susceptible to infection and vulnerable in case of disease [18,19]. Declining immune function substantially contributes to this higher susceptibility to develop or to increase prevalence and severity of some infectious diseases. The decline of immune defences with advanced age is well documented, especially the T-cell function decline in elderly person [13,20]. PBMC contain different cell types with distinct functions and corresponding gene expression profiles. The number of lymphocytes was significantly lower in aged patients in acute phase of infectious diseases. This could explain in part the changes of transcripts levels of gene specifically express in lymphocytes. However, the decrease of lymphocyte number was less marked than the large decrease of transcript levels of CD28, CD69 and LCK. In this study, we tried to identify transcriptomic biomarkers of the acute phase of infections in elderly patients. Healthy aged volunteers were used as control group, with the same mean age, in order to avoid age-related change of expression. Healthy aged probands gave blood and aged patients with infectious diseases gave blood twice, once in acute and once in convalescence phases. In the acute phase, a typical acute phase response was observed with higher levels of CRP, fibrinogen, D-dimers, leukocytes, neutrophils, IL-6 and TNF-α and lower level of albumin, pre-albumin and lymphocytes, as classically described in the acute phase response syndrome [21,22].

In this work, we searched for transcriptomic markers involved in stress response and immunosenescence, more specially focusing the T-cell compartments, and differentially abundant in the PBMC of old patients hospitalized due to acute infectious disease. Among the 82 transcripts eligible for data mining, 24 transcripts were differentially abundant with 15 more abundant and 9 less abundant in the PBMC of the infectious patients compared to the healthy aged probands. The transcript species whose abundance increased are known to be involved in oxidative stress, pro-inflammatory and anti-inflammatory response. The transcript species whose abundance decreased are involved in T-cell functions. At convalescence phase, 7 of these 24 transcripts came back to the normal values of the healthy aged probands (day 7-10), 12 significantly tended to come back to normal values while another 5 did not (DDIT3, EGR3, PRDX6, GPX1 and PSMD1).

Acute phase response is associated with increased oxidative stress. The transcript species of HMOX1, PRDX6, GPX1 and SOD2 were more abundant in PBMC of infected aged patients. HMOX1 gene encodes heme oxygenase, which is associated with oxidative stress in many cell types such as fibroblasts [23] and involved in stress response [24-27]. PRDX6 is an antioxidant enzyme overexpressed in stress-induced premature senescence [28]. GPX1 and SOD2 are major antioxidant enzymes [29,30] localised in the mitochondria (GPX1 is also found in the cytoplasm). The increase of HMOX1, PRDX6, GPX1 and SOD2 transcript species in PBMC of aged patients in acute phase might be a compensatory mechanism to cope with increased oxidative stress and damage in acute phase response. We compared previously the level of HMOX1 transcript between healthy young and healthy aged probands and found 1.46 fold increase in the aged probands [13]. The cumulated increase from healthy young probands to aged patients in acute phase represented a 2.25 fold increase (Table 3). As for PRDX6, the 23% decrease between aged and young healthy probands was exactly counterbalanced by the alteration observed between old healthy probands and aged patients in acute phase. Nothing is known on the induct ability of PRDX6 in young persons affected by sepsis.

**Table 3:** Ratios of abundance of seven transcripts between healthy aged (HA) and healthy young (HY) probands, between Aged patients with Infectious diseases in Acute phase (AIA) and HA and between AIA and HY.

Gene Symbol	HA/HY	AIA/HA	AIA/HY
CD28	0.62	0.61	0.39
CD69	0.70	0.44	0.33
LCK	0.77	0.58	0.45
			
CTSD	1.37	1.5	2.00
HMOX1	1.46	1.57	2.15
TNFRSF1A	1.68	1.45	2.33
			
PRDX6	0.77	1.23	1.01

These seven transcripts were found to be differentially abundant between PBMC of HA and HY [13].

The acute phase response is frequently associated with the activation of the inflammatory/anti-inflammatory response. There was an increase of the transcript abundance of the following cytokine receptors in infected aged patients: IL1R2, IL10RB, IL1R1 and TNFRSF1A. Among the 24 differentially transcripts identified here in acute phase, the abundance of IL1R2 transcript displayed the most important expression in acutely aged patients with infectious diseases (Fig 2). IL1R2, also known as CD121b, acts as a decoy receptor that inhibits the activity of its ligands [31] and inhibits IL-1 activity by acting as a decoy target for IL-1 [32]. Similarly the abundance of TNFRSF1A transcript was increased. Previous study showed that cytokine antagonist IL-1RA is elevated in plasma of aged patients and IL-2 production in vitro was lower with urinary tract infections compared to healthy aged [33]. Their results suggested that cytokine antagonists reduce IL-2 production and the capability of T cells to proliferate, thereby inhibiting responses in the elderly. Healthy aged also showed prolonged inflammatory activity in response to endotoxemia in vivo compared to young subjects. This phenomenon is probably due to changes in intensity to feed-back-inhibitory mechanisms or to an imbalance between the inflammatory and anti-inflammatory response [34]. The receptor IL-10RB for IL-10 (which binds also IL-22) serves as an accessory chain essential for the active IL-10 receptor complex and to initiate IL-10 induced signal transduction events. The abundance of the transcript of IL10-RB increased in acute phase in the aged patients. IL-10 is an anti-inflammatory cytokine. IL-10 release was also increased (Table 1) in aged patients in acute condition. Previous in vitro studies showed that IL-10 exerts an inhibitory effect on the expression of pro-inflammatory cytokines in PBMCs [35,36]. Production of IL-10 also suppresses the adaptive T-cell response [37]. IL-10 enhanced production is believed to be an anti-inflammatory regulatory mechanism by which the ageing immune system auto-regulates the potential damage caused by its excessive activation [38]. This modulates the intracellular signaling process to favour the production of anti-inflammatory rather than pro-inflammatory agents [39,40]. Taken together, these results suggest an imbalance between the inflammatory (increased release of IL-6) and anti-inflammatory (increased release of IL-10) responses in acute phase of infection in aged patients. In addition to the immunological and biochemical age-related changes, this might lead to a defective inflammatory response and then a defective responsiveness of aged patient to the pathogenic challenge [41].

The decline of the immune function is supported by many epidemiologic and clinical observations [42,43]. In this work, we observed a decreased abundance of CD28, CD69 and LCK mRNA in acutely infected aged patients, compared to healthy aged. The comparison of the levels of CD28, CD69 and LCK transcripts between healthy aged and healthy young probands previously showed a respective 38%, 30% and 23% decrease in the aged probands [13]. Compared to healthy young probands, the difference was respectively to 61%, 67% and 55% (Table 3). These dramatic alterations, if confirmed at protein levels, might really affect T-cell activation. CD28 and LCK molecules indicate TCR (T cell receptor) signaling pathways. Interaction of the TCR with an antigen induces a rapid early cascade of intracellular signaling events that lead to T-cell activation. Nevertheless, the binding of TCR to antigen alone is insufficient to drive immune reaction. Co-stimulation is a critical step in fully activating T cells. The accumulation of CD28-effector T-cells has been shown to accompany an impaired response to influenza vaccination [44]. TCR engagement recruits LCK and FYN, which are protein kinases initiating the TCR signaling pathway [45]. Decreased abundance of CD28 and LCK mRNA indicates that early signaling events following antigen stimulation are altered in T lymphocytes. This hypothesis of lowered activation was reinforced by the 66% decrease of the mRNA level of early activation surface markers CD69 found in comparison with healthy aged subjects (Table 2). These changes in the T-cell activation mechanisms probably explain the lower efficiency of vaccination procedure and the vulnerability in case of disease in old persons, where TCR antigen recognition and T-cell activation are primary phenomenon involved as well as higher infectious risk when involving adaptive immune system.

We observed previously a 29% decrease of the number of lymphocytes in the PBMC in aged patients. The decrease of the mRNA level of CD28, CD69 or LCK might be also linked in part to the decrease of the number of lymphocytes. Lastly, we also observed previously a 1.37 fold increase of the abundance of CTSD transcript in healthy aged, compared with healthy young probands [13]. The comparison of the level of CTSD transcript showed a 2 fold increase between healthy young probands and acutely aged patients with infectious diseases (Table 3). CTSD gene codes for cathepsin D, a lysosomal protease involved in physiological protein degradation [46] and supposed to play role in antigen processing [47].

In conclusion, the acute phase response to infections is altered in elderly patients. Decreased abundance of transcript species associated with T-cell functions and increased abundances of transcript species associated with inflammatory and anti-inflammatory responses in acute phase suggest a decline of the immune function and an impaired inflammatory responses. A better insight into the basic mechanisms of immune dysfunction, changes of inflammatory response and oxidative stress that occur in acute phase on aged patients will help to intervene and thereby ensure a better protection of the vulnerable elderly population from disease, subsequent loss of independence, and death.

It would be interesting to determine the abundance of the corresponding proteins and to know whether the abundance of all the transcripts of classes 2 and 3 returns to normal values later than day 7-10 or not. This would require following the outcome and obtaining blood of the patients for several weeks/months after hospitalization, which is out of the scope of this study and poses potential ethical problems.

## Abbreviations

CRP: C-reactive protein; IFN-γ: interferon-gamma; IL-6: interleukin-6; IL-10: interleukin-10; PBMC: peripheral blood mononuclear cells; PMT: photo-multiplier; RT-qPCR: retrotranscription quantitative polymerase chain reaction; TCR: T cell receptor; TNF-α: tumor necrosis factor-alpha.

## Competing interests

The authors declare no conflicts of interest. This paper was not mentioned in any meetings or congress.

## Authors' contributions

TKDV, PG, FCD, CS, HJM and OT conceived and designed the experiments. TKDV, PG and OT performed the experiments. TKDV, PG, MDSH and OT analyzed the data. TKDV, PG, GM, MDSH, CS, VG, HJM and OT contributed reagents/materials/analysis tools. TKDV, MDSH, FCD, CS, and OT wrote the paper. All authors read and approved the final manuscript.

## Supplementary Material

Additional file 1 **Table S1 - Gene symbol, gene name and GenBank accession number of all transcripts which abundance was determined in this study**. A specific technology of low-density DNA arrays was used to study, in peripheral blood mononuclear cells, the relative abundance of 135 transcripts species involved in immunosenescence, inflammation and stress response plus 13 housekeeping genes. The list of the corresponding genes in given in this additional file. Click here for file
